# “No one takes it seriously”: U.S. military personnel with gambling concerns: recommendations for expanding treatment and public health efforts

**DOI:** 10.3389/fpubh.2026.1745979

**Published:** 2026-07-23

**Authors:** Shane W. Kraus, Bailey M. Way, Karen A. Valle Frias, Frankie A. Nieblas, Naama Glaser-Guy, Jenia Kelner, Belle Gavriel-Fried

**Affiliations:** 1 Department of Psychology, University of Nevada, Las Vegas, NV, United States; 2 The Bob Shapell School of Social Work, Tel Aviv University, Tel Aviv-Yafo, Israel

**Keywords:** gambling disorder, recommendations, treatment seeking, U.S. military, veterans

## Abstract

**Introduction:**

Gambling is increasingly recognized as a significant public health issue, with negative consequences for financial stability, social well-being, and overall health. Growing evidence indicates that U.S. military personnel and veterans are a high-risk group for gambling-related problems, including gambling disorder (GD). Despite the challenges posed by GD, military personnel rarely seek treatment or disclose their struggles. The present study aimed to identify and develop treatment and policy recommendations to improve help-seeking and disclosure among U.S. military personnel with lived experience of GD.

**Methods:**

Semi-structured interview were conducted with 28 U.S. veterans and active-duty service members who reported current or past experiences with GD.

**Results:**

Content analyses revealed four themes: (1) “*Increase awareness,*” reflecting a need to raise awareness of gambling as a problem through gambling education, financial literacy training, and screening for GD; (2) “*Open door policy,*” highlighting the need for a safe and confidential environment to disclose gambling concerns; (3) “*Eliminate gambling and increase leisure,*” demonstrating a need to reduce access to gambling and promote healthy leisure activities within the military; and (4) “*Increase resources for treatment,*” emphasizing the need for GD-specific treatment programs, greater awareness of available treatment options, access to on-base specialists, and peer support.

**Discussion:**

These findings reveal treatment needs among U.S. military populations with GD and point to structural changes the U.S. military should adopt to reduce GD, promote help-seeking, and strengthen overall health, readiness, and well-being.

## Introduction

Public health researchers are increasingly recognizing gambling as a significant public health concern (1). Scholars have begun expressing concern about the rapid expansion of legalized gambling avenues across the United States ([U.S.]; e.g., sports betting, online casinos), as these are associated with adverse health consequences, including gambling disorder (GD) (2, 3). GD is an addictive disorder characterized by a persistent pattern of gambling that leads to distress and impairment, whereas problem gambling (PG) is a broader term for harmful gambling behaviors that do not necessarily meet the diagnostic threshold for GD. Although some policies exist to address gambling problems, they generally lack a structural public health framework, and tend to focus disproportionately on individual pathology (4). Other scholars have questioned industry involvement in gambling policy and research, warning that their presence may normalize gambling, minimize harm, and exploit vulnerable populations (5).

In the U.S., the 12-month prevalence of GD is estimated at 0.2–0.3% (6), though rates vary widely across different populations. Recent research efforts have identified a multitude of populations at risk for PG, including men, youth, disadvantaged socioeconomic groups, individuals with marginalized identities, individuals with co-occurring mental health conditions, and individuals with a family history of gambling problems (7–12). Research shows that U.S. military populations (i.e., service members and veterans) are uniquely vulnerable to PG and experience disproportionately more gambling-related consequences (7). Research suggests a higher prevalence of PG among veterans in both the U.S. and United Kingdom (UK) compared to civilians (7, 13), with some evidence indicating that female veterans may be particularly vulnerable (14, 15). Service members and veterans with PG face high rates of psychiatric comorbidity, including substance use disorders, mood disorders, personality disorders, and posttraumatic stress disorder (7, 8, 16, 17). Additionally, PG may be a unique risk factor for suicide in veterans worldwide, as GD appears to predict suicidality, and veterans with GD often report having attempted suicide at some point in their life (18–20).

Despite these disparities, little is known about how structural and organizational elements affect military communities. Military personnel and veterans face unique internal and structural barriers to engaging in mental health treatment, such as inaccessibility (21), financial and transportation difficulties (22), and inadequate provision of care (23, 24). Qualitative studies have highlighted that treatment-related stigma and the normalization of gambling remain central barriers to help-seeking among service members and veterans (21, 25). For example, a study of UK Armed Forces personnel identified secrecy surrounding gambling, fear of reputational damage, and reluctance to disclose concerns as major barriers to early identification and help-seeking (25). Participants also reported dissatisfaction with available treatments and emphasized the importance of informal, peer, and anonymous support. Suggested solutions included early screening, educational courses, and mandatory training. However, the study was limited by its focus on mostly participants with low or recovered PG symptoms, raising concerns about representativeness and its ability to capture the perspectives of those actively struggling with GD.

The Department of Defense (DoD) recently issued guidelines for the prevention and treatment of substance use problems and GD within the U.S. military (26). These include general prevention screening, encouraging help-seeking, implementing evidence-based treatments, providing annual training, and developing community prevention programs. However, while these guidelines mark progress, they remain insufficient to fully address gambling-related harm in the military, particularly given that individuals with lived experience did not directly contribute to their conceptualization. Incorporating such perspectives is essential, as service members with firsthand experience are uniquely positioned to highlight contextual factors, such as the widespread accessibility of gambling opportunities (e.g., slot machine parlors on overseas bases) and a military culture that often normalizes gambling as harmless recreation (21, 25). We posit that such unfettered access to slot machines, combined with cultural normalization, creates a high-risk environment for gambling-related harm.

Presently, no U.S. federal legislation provides specific support for GD. Several congressional bills have been introduced, but none have become law. For example, the Gambling Addiction Prevention Act (27) was introduced in 2019 to require the DoD to implement programs for the prevention, education, and treatment of GD, but it was not enacted. Recently, the Gambling Addiction Recovery, Investment, and Treatment Act (28) was introduced to mandate national funding for prevention and treatment, though it remains under committee review.

Scholars have recommended strategies in the general population such as restricting access to commercial gambling (29) and educational interventions, which have been shown to increase help-seeking for mental health concerns among service members (30). However, effective structural changes within the U.S. military remain unclear, and the impact of these interventions on GD has also not been examined. The current study is part of a larger project that demonstrated that military service members and veterans experienced unique barriers to PG intervention (21). Although strategies to reduce gambling harm in the general population are emerging (25, 29), there is little guidance on prevention and intervention approaches specific to U.S. military service members with lived experience. Therefore, we sought to amplify the perspective of military service members and veterans with GD to identify and formulate treatment and policy recommendations for the U.S. Armed Forces and veterans. Implementing these recommendations would help to increase help seeking and disclosure among this group. Our research question is: what recommendations do veterans and active duty suggest for increasing help seeking and disclosure for gambling concerns in the military?

## Method

### Methodological approaches

A qualitative descriptive approach was employed, as it is well-suited for addressing policy-relevant questions (31, 32) and is commonly applied in health care research. As a pragmatic methodology, qualitative descriptive research offers rich, straightforward accounts of participants’ perceptions and experiences related to the research question (33).

### Procedure and participants

A purposive sampling was utilized using a combination of criterion-based case selection and maximum variation strategies (34). Eligibility criteria required participants to be at least 18 years of age, either currently meet diagnostic criteria for GD or have met diagnostic criteria for GD in the past 10 years determined by endorsing at least four symptoms of the GD criteria outlined in the DSM-5 (6) and be either currently serving in or formally discharged from the U.S. Armed Forces.

This resulted in 28 participants who met criteria for GD in the past 10 years. Twenty-two of these participants currently met diagnostic criteria for GD (classified: non-recovered), and 6 were in remission (classified: recovered). Overall, the sample was composed of 28 U.S. service members or veterans, ranging in ages from 27 to 73 (*M_age_* = 46.5). The sample included 15 White-identified participants, 6 Black-identified participants, and 7 Latino, Asian, Native American, or Multiracial-identified participants. Most participants identified as men (*n* = 20; 71.4%), and 8 participants identified as women (28.6%); no participants identified with any other gender (e.g., nonbinary). Participants included veterans (*n* = 24; 85.7%) as well as active service members (*n* = 4; 14.3%). Participants represented multiple branches of the military including the Air Force (*n* = 10), Navy (*n* = 5), Marine Corps (*n* = 4), and Army (*n* = 9).

We recruited participants through flyers, social media platforms, and by engaging with influential figures in the veteran community. Furthermore, organizations supporting veterans and service members (e.g., clinicians, medical centers) were contacted to promote the study. Recruitment efforts continued nationwide until saturation was reached (35).

Eligible participants were first screened via the phone with a screening intake conducted by a clinical psychology doctoral student. For those who met study inclusion criteria, semi-structured interviews through Zoom meetings were conducted by two clinical psychology doctoral students after obtaining informed consent. A licensed clinical psychologist supervised all initial screenings and interviews.

Participants were asked two broad questions to provide recommendations to improve disclosure of gambling problems and help-seeking among military personnel (1) *What changes would you recommend to the U.S. Armed Forces to increase help-seeking for treatment for gambling problems among military personnel struggling with gambling problems?* and (2) *What changes would you recommend to the U.S. Armed Forces to increase disclosure of gambling problems among military personnel?* The interviews were recorded and transcribed verbatim. The interviews were conducted from August 2023 to August 2024, and participants were compensated with a $75 gift card. This project was approved by the Institutional Review Board at author’s university (University of Nevada, Las Vegas #2022-650). All names used in the findings are pseudonyms.

### Data analysis and trustworthiness

An inductive content analysis was conducted following these steps (36). First all interviews were read thoroughly to promote familiarity. Five interviews capturing variation in sociodemographic characteristics and military backgrounds were selected. Next, two team members (KVF and FN) independently coded content related to participants’ recommendations inductively. This process resulted in an initial codebook of 18 codes (e.g., more screening of people in power positions, online counseling, confidential counseling, etc.). These initial codes were critically reviewed and discussed by the full research team (KVF, FN, BMW, SWK, BGF, NGG, JK), a process that enhanced the confirmability (37, 38). Discrepancies were resolved and examined until consensus was reached. At the end of this stage several codes were refined, Subsequently, the remaining interviews were coded using the refined codebook by the two team members (KVF and FN). Any relevant segments that did not align with existing codes were inductively coded and added as new codes (e.g., *more opportunities for leisure time, peer support, etc.*) which resulted in 30 codes (e.g., *on base gambling counselor, increase resources for gambling, take care of the soldiers*, etc.). Again, any disagreement related to the labeling of these codes were resolved through team discussion, and a few codes were refined or split (e.g., *broad awareness* code was split *into (1) awareness of how to seek help, (2) awareness of the gambling problem, and (3) advertisement*) which resulted in 36 codes.

Upon completion of this process, the other team members (BGF, SWK, BMW, JK, NGG) reviewed the full set of codes and approved them. The codes were then grouped and formulated into 12 categories according to similarities and differences which were further conceptualized into four core themes. These themes represent the key recommendations offered by military veterans and service members regarding strategies for improvement, treatment, and prevention of gambling-related issues.

The analytic process was conducted using MAXQDA software. The software facilitated systematic data organization, coding, and documentation of analytic decisions, thereby enhancing the study’s dependability (38). Finally, to enhance the transferability, the findings present rich and thick quotes (39). Table 1 presents the themes and categories.

**Table 1:** Illustration of the recommendations among veterans and service members: Theme, categories, codes, and exemplar quotes.

Themes	Categories	Exemplar Codes	Exemplar quote
Increase awareness	“Increase awareness of the problem”	Broad awareness Awareness of the gambling problem	**Sherry**: *They should discuss it more because it’s a very silent disease, gambling use disorder […]*
	“Mandatory briefings on gambling”	Briefings on gambling	**Doug**: *I would have mandatory briefing counseling you know, you got to come to this and listen, like you get briefings all the time in the military. I would make it mandatory […]*
	“Increase screening for gambling problems”	Screening for gambling problems Personalized plan	**Brayden**: *But as a veteran, I think it’s important that they maybe do a separate questionnaire about gambling, you know […]*
	“We should be taught how to handle money”	Educate everyone Education on personal finance and money handling	**Jared**: *[…] we should be taught about these things […] how to handle money in the military, they do not tell you how to handle money […] They do not teach you anything about finances […]*
Open door policy	“Create a safe environment to talk about gambling without repercussions”	Permission to talk Open door policy without repercussions	**Doug**: *But the second thing would be is open door policy to go in and say, ‘Hey, I’ve got a problem.” You know, without repercussions […] So there has to be an open door. No harm, no fouls, you come to me and say you need help for gambling.*
	“Let us be private, confidentiality assured”	Importance of confidentiality for disclosure Confidential counseling/therapy/treatment	**Elliot**: *Let us be private, confidentiality assured. And I think, I think it will drastically improve the number of people seeking help*.
Eliminate gambling and increase leisure	“Eliminate access”	Eliminate all forms of gambling Take slot machines out remove slots overseas	**Maria**: *[…] Stop creating more gambling things-more things for us to gamble on. We do not need more and more things to gamble on is what I mean. That’s what I’m trying to say. Do not create more access. That was my point. Do not create more access.*
	“Increase leisure”	More opportunities for leisure time	**Freya**: *And when they start actually take care of you, mentally, emotionally, give you these opportunities to […] distract yourself […] So, if they could, if they could provide more, more opportunities to enjoy your life outside the army.*
Increase resources for treatment	“More treatment programs for gambling disorder”	Increase resources for gambling New programs for gambling	**Madison**: *I definitely think I would say something similar that I think there should be some resources and support groups out there for veterans that struggle with gambling […]*
	“Make more resources available to let people know about these resources”	Awareness of how to seek help Websites Advertisements	**David**: *I think they should advertise more, like, hey, if you guys have this problem, you can go here or here, here, you know, I think they have all these resources available, but they do not ever tell anybody about them.*
	“On base gambling specialist”	On base gambling counselor	**Doug**: *And I think that […] there needs to be a place on base that you can go to. You know, somebody dedicated just for that gambling addiction […] and having somebody on that base that is trained, that has some kind of knowledge of gambling addiction and the addictive behaviors.*
	“Peer support groups”	More peer support Battle buddies	**Maria**: *Peers should know what to look for in each other, signs, you know, and we talk about it […] just have people trained to identify [.] you could refer them to the proper channels […]*

## Findings

Participants revealed an overarching problem with the way the military system handles individuals with gambling problems. Many participants indicated that there is profound failure to recognize gambling as a problem. For example, Mark (51, White, man, non-recovered, veteran) expressed that *“no one takes it seriously”* whilst Ryan (73, White and Middle Eastern, man, non-recovered, veteran) indicated that*: “they do not do enough in terms of this addiction at all anywhere in the US.”* The interviewees often contrasted gambling and substance use and further expressed gambling problems are not taken as seriously as substance use problems. They provided many recommendations to address this deficiency within the U.S. military system, including addressing the lack of awareness, discussion, and resources surrounding gambling problems in the military. Their recommendations for improving help-seeking and disclosure were intertwined with criticism, frustration, and disappointment toward the military system. The following four themes were identified: (1) increase awareness, (2) open door policy, (3) eliminate gambling and increase leisure, and (4) increase resources for treatment. These themes highlight the clear recommendations for the U.S. military to take gambling problems seriously and provide adequate support to encourage disclosure and help-seeking.

### Increase awareness

Participants called for changes that should be made to increase awareness of gambling problems. The participants discussed various educational and screening methods that should be implemented to increase awareness to gambling problems in the military. Some participants suggested increasing education on risk factors of PG and personal finance to improve prevention whereas others recommended implementing specific methods to identify those who need help.

#### Increase awareness of the problem

Participants highlighted the necessity to increase awareness of gambling as a problem in the military system. Specifically, they recommended more education for service members on the invisible symptoms of GD and its warning signs. Elliot (28, Black, man, non-recovered, service member) presented education as pivotal to identifying and preventing gambling problems:

Educate the masses on how to identify addiction because I believe if as a group, we had this education that we are addicted to gambling by now, that would help. I think that will be like a major game change, and it will help out a lot and maybe educate people in the process of avoiding the addiction route. And also steps on how to seek help about gambling.

Sherry (39, Black, woman, non-recovered, veteran) also discussed the need for awareness of the invisible signs of gambling addiction:

They should discuss it more because it's a very silent disease, gambling use disorder. It's very easy to hide until you can't hide it no more. […] It's very silent, you know, you can't, you don't see it on the person like you do with drugs or alcohol and stuff.

Cristina (54, Black, woman, non-recovered, veteran) also recommended information on compulsive behaviors should be standardized and provided earlier: “I think in boot camp, there should be some information covered on compulsive behaviors and seeking help.”

#### Mandatory briefings on gambling

Some participants suggested including briefings as a method for increasing awareness. Interviewees recommended there should be briefings specifically on gambling problems, and some suggested that these should be mandatory. Doug (59, White, man, non-recovered, veteran) recommended information on the dangers of gambling should be disseminated through mandatory briefing counseling:

I would have mandatory briefing counseling you know, you got to come to this and listen, like you get briefings all the time in the military. I would make it mandatory. ‘Hey, you know it's part of your in-processing when you get to a new base. You gotta go to a, you know, gambling, you know, whatever meeting.’ […] Have mandatory briefings on gambling and dangers of gambling.

#### Increase screening for gambling problems

Interviewees emphasized the need for screening methods (i.e., questionnaires, evaluations, checklists) that focus on identifying PG among all service members (e.g., sergeants, NCOs, healthcare professionals) before, during, and after their service.

Brayden (57, White, man, non-recovered, veteran) recommended incorporating questionnaires that screen specifically for GD:

But as a veteran, I think it's important that they maybe do a separate questionnaire about gambling, you know. […] And I think, they should maybe structure something like that for like a gambling questionnaire, because it's another addiction.

Likewise, Cody (29, Black, man, non-recovered, veteran) suggested regular check-ins, GD specialists, and screenings for GD would aid with early detection:

If they could like, you know, establish protocols that can help identify, you know, individuals or people who are in the military specialize in gambling-related issues, you know, like doing things like regular check-ins and assessments, yeah, like I think the gambling problem can be like identified earlier, that could be a little better, yeah.

Additionally, Robert (35, White, man, recovered, veteran) recommended that service members should be screened prior to leaving the military to provide personalized help and resources:

We really need to talk about gambling. […] Every soldier should get a personalized health care or personalized out processing experience. […] There should be a one general screening […] So, it’s the same thing with drinking.

#### We should be taught how to handle money

Another aspect mentioned by the participants related to awareness is related to the financial education. Participants expressed the importance of receiving education on personal finance, money handling, and making good financial decisions while in the military so they would have the knowledge and awareness needed to manage their finance. Jack (38, Asian, man, non-recovered, veteran) recommended specific education on personal finance to help prevent gambling problems:

So maybe just educate everyone to have, like a baseline level of knowledge about personal financing, handling money, and credit and all that stuff. Then I think they would not have to go through like struggling up until the point they get out and then realizing what they have been doing.

Jared (33, Latino and Asian, man, non-recovered, veteran) was frustrated by the lack of education on finance he received while in the military and recommended trainings to teach service members how to handle money:

We should be taught about these things, how to handle money in the military, they do not tell you how to handle money. […] They do not teach you anything about finances, not, one point, in that my whole fucking four years of active duty, did they ever give me one finance class. Not one finance class. Not one!

### Open door policy

The second theme focuses on recommendations centered around creating a safe and private environment where service members are encouraged to seek help and discuss their gambling problems. This theme reflects the critical importance of safe and confidential discussion without the fear or risk of consequences.

#### Create a safe environment to talk about gambling without repercussions

To increase disclosure of gambling problems, participants recommended creating a safe and comfortable environment where service members are encouraged to discuss their experiences with gambling problems. Doug (59, White, man, non-recovered) highlighted that many individuals will come forward once the problems have become severe, and he recommended having a non-punitive open door policy to disclose gambling issues:

But the second thing would be is open door policy to go in and say, ‘Hey, I’ve got a problem.’ You know, without repercussions. You know, a lot of times when they, when somebody will go in and admit they have a problem, they have already hit the rock bottom, they could not pay their bills or whatever, their funds getting shut off. And to me, if you punish somebody, if they come to you for help, even when they are at the rock bottom, you are doing them wrong because they are not gonna go for any more help. You know, because now you are afraid of reprisals. So there has to be an open door. No harm, no fouls, you come to me and say you need help for gambling.

Similarly, Clarie (56, Black, woman, non-recovered, veteran) recommended reassuring service members that if they disclose gambling issues they will not be punished, thereby increasing the number of people who will seek help:

I think if they could give people the reassurance that if you come forward we will not just kick you out of the military without helping you. If you give people that reassurance that we’ll help you and then see if you are still fit. But just not kick you out and then you are just with no means of income, no way of supporting yourself, you know. I think that would be a better route if they could just help you and you know reassess from there. But just you come forward and it’s like, ‘Oh, you are just getting kicked out.’ I do not think that’s right. I do not think that’s right.

Madison (42, White, woman, non-recovered, veteran) recommended increasing the comfort levels around discussion of gambling problems: “Let people know that it’s okay, you know, bring that comfort level to let them know it’s okay to talk about that.”

Cristina (54, Black, woman, non-recovered, veteran) also recommended normalizing language and discussions around help-seeking for gambling problems: “[…] I think that if it’s not normalized to seek help, people aren’t going to seek help. They’re not going to seek help until it’s out of control.”

Similarly, Mason (55, Latino, man, non-recovered, veteran) also emphasized the importance of creating a safe environment where service members can talk about their gambling issues: “If something gets identified early. Get to it, jump to it, you know, create a safe environment for them to be able to come on in one on one, you know. Just to talk to you.”

#### Let us be private, confidentiality assured

Interviewees recommended increasing confidential measures as another strategy to increase the disclosure of gambling issues among service members. They suggested that confidentiality should be assured in reporting and treatment. Elliot (28, Black, man, non-recovered, service member) suggested ensuring confidentiality to increase the number of people who seek help: “Let us be private, confidentiality assured. And I think, I think it will drastically improve the number of people seeking help.”

To further increase disclosure, participants recommended ensuring confidential counseling. Specifically, they recommended that the military create policies for mental health workers to not disclose gambling issues to superiors. Edward (51, Asian/Pacific Islander, man, non-recovered, veteran) suggested having telehealth options to increase confidentiality, thus allowing service members to seek help and have confidential options for treatment.

Basically, give veterans or give active duty, military the option to seek treatment without exposing it to their command. Command is their bosses in English. […] So, whatever you are doing, right, advertise it on the base, right? If you have a problem with it, you know, not you, but whoever right, if you have a problem with addiction, this is here, it’s confidential. We can meet by Zoom, whatever, and it gives them options. That’s the easiest way for people so they do not. It’s confidential. And you can and you do not have to actually go to the place, right? And it gives you option to get treatment without anyone finding out.

### Eliminate gambling and increase leisure

The third theme focuses on recommendations that relate to the environmental and cultural aspects of the military. This theme relies on the critical view of the participants regarding easy access to gambling while serving in the military. Specifically, the interviewees expressed frustration with military policies that enable easy access to gambling venues. They emphasized the harmful effects of the combination of easy access to gambling and military-related stress. The participants recommended eliminating access to gambling and increasing opportunities for more leisure time to engage in self-care outside of the military.

#### Eliminate access

Many participants indicated that access to gambling should be restricted. For example, Doug (59, White, man, non-recovered, veteran) asserted that the military should: “get rid of the machines off of the bases.” Similarly, Maria (50, White and Filipino, woman, recovered, veteran) recommended that access to gambling should not be enabled:

Stop like- stop the lotteries like you do not like, stop! Stop-stop popping up with more things to… Stop creating more gambling things-more things for us to gamble on. We do not need more and more things to gamble on is what I mean. That’s what I’m trying to say. Do not create more access. That was my point. Do not create more access.

In a frustrated tone, Cristina (54, Black, woman, non-recovered, veteran) expressed confusion regarding the easy accessibility of gambling on military bases:

I do not understand why things that hurt us and can become addictive are on base. Like I do not get that. Like, they have to know the correlation between gambling and addiction. They’re so much smarter than we are. So, I’m sure that they know that. Why else would they put the casino on bases? Why else would you allow younger people to drink alcohol? It was like the culture was what it was. And it wasn’t healthy. Like, I do not think those things are needed to produce good soldiers.

#### Increase leisure

While most interviewees advocated for eliminating gambling, one participant targeted prevention by suggesting more opportunities to engage in pleasurable, meaningful, and healthy activities (e.g., time with their family) outside of the military. Specifically, Freya (31, White, woman, non-recovered, service member) recommended increasing leisure time through off-site trips that will provide the chance for service members to de-stress and engage with their loved ones:

And when they start actually take care of you, mentally, emotionally, give you these opportunities to go for the trip or they can have you this operation to take a, you know, four day pass and actually go and travel, see your friends, family, distract yourself, get out far away because you know you have only two days and one–one way to Houston’s only like three hours and then you coming back and you feel tired already and just like not enough. So, if they could, if they could provide more, more opportunities to enjoy your life outside the army.

### Increase resources for treatment

The fourth theme centered around the need to increase resources for GD support in the military. The participants overwhelmingly indicated a lack of resources for GD support, and this was often contrasted with more accessible support for substance use problems. The interviewees recommended increasing resources specifically for GD by creating new treatment programs for gambling, including programs for women, having specialized mental health professionals on bases, and increasing peer support programs for gambling problems.

#### Make more resources available to let people know about these resources

Many participants suggested steps should be implemented to improve the awareness and accessibility of available resources. Freya (31, White, woman, non-recovered, service member) suggested providing information on resources and making the information easily accessible:

Make more resources available to let people know about these resources because in my digit station there were no real information about that, like if you, yes, if you have only drug addiction because you have this, you at that basically, so they would find out about this easily.

Some participants recommended advertising resources for gambling treatment. Kaleb (37, White, man, non-recovered, veteran) advised creating more advertisements discussing available resources for seeking help with gambling. He contrasted the lack of advertisements for gambling support with the much larger number of advertisements that encourage gambling:

I mean, I know they have 1–800 numbers, maybe a little more advertising in the counseling area, you know, hey, if you feel like you have a problem call us, talk to us, cause I’ve seen a lot more gambling ads obviously ESPN Bet, FanDuel, Draft Kings, William Hill, so on and so forth, advertisements for hey gamble with us, but I have not seen the advertisements for, ‘Hey, if you have a trouble gambling.’ I feel like it’s just advertisements on one side are on the rise.

David (45, White, man, recovered, veteran) also advised increasing advertisements for resources in the military: “I think they should advertise more, like, hey, if you guys have this problem, you can go here or here, here, you know, I think they have all these resources available, but they do not ever tell anybody about them.”

Similarly, Sherry (39, Black, woman, non-recovered, veteran) suggested creating an online website showcasing available resources for PG support:

Maybe a website with like all of the resources like everything because you’ll find plenty of websites. But they’ll be local specific like to that state or just to this demographic or you have to have insurance or, you know, and you have to find one, like out of look that you fit in and then that can help you type of thing or access to it. So maybe like a national website or something.

#### More treatment programs for gambling disorder

Participants indicated that there should be programs specifically for the treatment of GD. Madison (42, White, woman, non-recovered, veteran) suggested increasing resources and support for veterans who struggle with gambling problems:

I definitely think I would say something similar that I think there should be some resources and support groups out there for veterans that struggle with gambling. They have a lot of things out there for veterans, but I do not think that there’s anything related to gambling addiction. […] I’m thinking it may be warranted to consider having some sort of support or resources for those veterans that are struggling with addiction other than alcohol, or those that things quote unquote, that are, you know, normal are more common addictions.

Clarie (56, Black, woman, non-recovered, veteran) suggested creating new programs to help women with gambling problems:

I think there needs to be more, I do think that there needs to be more programs for women. Most of the programs in the Veterans Affairs are geared toward men because there’s I guess there’s more male veterans, but there’s more women joining the military than ever. There are more female veterans than ever, and I think there needs to be more programs, and more help for female veterans.

#### On base gambling specialist

Interviewees also recommended that the military should make GD treatment more accessible. For instance, Doug (59, White, man, non-recovered, veteran) recommended that every base should have access to a mental health professional specialized in GD treatment:

And I think that every base, you know, there’s they have drug, you know, counseling on base, they have, you know, alcohol classes on base. There needs to be a place on base that you can go to. You know, somebody dedicated just for that gambling addiction. I mean, they can have, yeah, be in charge of the gambling, alcohol in a couple of things, but you have to have somebody there with knowledge of gambling addictions. […] And having somebody on that base that is trained, that has some kind of knowledge of gambling addiction and the addictive behaviors.

Similarly, Kaleb (37, White, man, non-recovered, veteran) suggested making treatment more accessible by having a therapist assigned to each squadron:

I do not know just the accessibility. […] Like a therapist assigned to each squadron like an in-house therapist cause you know your squadrons are spread out all over the base, and if you want to talk to somebody, you gotta go to mental health. Well, that’s one building, so maybe creating like a mental health advocate that is assigned to each squadron. They can say, ‘Hey, you know you got stuff going on.” Let us get you over to mental health, or let us get you in touch with the right people.’ I think that’s probably the best way.

Similarly, Robert (35, White, man, recovered, veteran) recommended that training supervisors to be responsible for recognizing signs of GD in their team members:

[…] Sergeants, NCOs, noncommissioned officers should know their subordinates and should identify, they should go through training of recognizing key aspects of like identifiers.

#### Peer support groups

Interviewees suggested incorporating more peer support resources as a method for improving treatment, prevention, and support. For example, Grace (53, Multiracial, woman, non-recovered, veteran) recommended utilizing the Battle Buddies program to support service members with gambling concerns. She expressed that peers should support each other to foster open discussion on mental health:

You know, we talk about Battle Buddies a lot, but we do not really put it into practice very much. Like looking out for your fellow soldiers, if you see something. I hate that whole, if you see something, say something, but sometimes it can be a good thing, you know. So, you know, you were talking about having that camaraderie with other veterans and other soldiers. Whether they are on active duty or not. And I think that we could do a better job of looking out for each other.

In addition, Robert (35, White, man, recovered, veteran) suggested creating a mentorship program among service members, where more experienced members are paired with newer personnel to act as mentors:

There needs to be more like mentorships. Like, I really, I really like, I like the idea. I wish I had a mentor, transitioning mentoring, like maybe a year out, like a big brother, little brother kind of thing like you get with somebody that actually went through it all. Like if a government starts to tell you something, I just remember just in one ear out the other. But if another vet talked to me like, I’m like, wow, this is this actually might actually happen to me. So, I need to listen up. So maybe yeah, like a peer to peer.

Similarly, Maria (50, White and Filipino, woman, recovered, veteran) recommended having a squad leader that is trained to identify the signs of gambling disorder that can guide service members struggling to the proper resources:

Peers should know what to look for in each other, signs, you know, and we talk about it […] just have people trained to identify these like, just general knowledge of signs, you know, so that, you know you could refer them to the proper channels, and then also have these professionals available for counseling.

## Discussion

The present qualitative study amplifies the voices of service members and veterans experiencing GD in order to identify treatment and policy recommendations to improve disclosure and help-seeking within the U.S. military. Overall, we found participants emphasized that the U.S. military fails to adequately recognize gambling as a problem. Through interviewing, participants provided actionable strategies to address these shortcomings and expand support for those experiencing issues with GD. Below, we outline four themes that emerged, evaluate their feasibility, and suggest strategies for implementation in the context of existing U.S. military policies (implementation strategies organized by theme are summarized in Table 2).

**Table 2:** Themes, recommendations, and implementation strategies.

Theme	Recommendation	Implementation
Increase help-seeking		
Increase awareness	“Increase awareness of the problem”	Provide education on GD and the early signs of GD to military personnel, as well as the general population. This education will aid in destigmatization, as well as identification of GD by the individual and/or by their support system (i.e., co-workers, superiors, family, friends). Implement advertisement campaigns for GD signs.
	“Mandatory briefings on gambling”	Provide education training through formal mandatory briefings while in the military.
	“Increase screening for gambling problems”	Include regular screening for mental health concerns in annual medical doctor appointments, such as the BBGS (43). This regular screening should be conducted before, during, and after service.
	“We should be taught how to handle money”	Provide education on personnel finance, money handling, and making good financial decisions.
Open door policy	“Create a safe environment to talk about gambling”	Aid in the destigmatization of mental health concerns, especially GD, through education and safe spaces (e.g., superiors and counselors that will allow disclosure and help-seeking without punishment). Conduct annual trainings for mental health care providers in the military on ethics, how to mitigate role strain, and increase quality-enhancing behaviors for their clients and the military organization (e.g., seeking consultation when unsure).
	“Let us be private, confidentiality assured”	Similar to substance use disorder treatment, the creation of confidential laws to protect individuals experiencing GD to increase likelihood of disclosure and help-seeking. Ensure that service members are provided with clear confidentiality guidelines and expectations to increase help-seeking and disclosure
Prioritize accessibility of gambling treatment		
Eliminate gambling and increase leisure	“Eliminate access”	Remove slot machines from military bases. Remove access to online gambling, including sports betting avenues, and limit advertising related to gambling.
	“Increase leisure”	Provide other leisure activities. Provide more opportunities for military personnel to connect with family and friends outside of the military.
Increase resources for treatment	“More treatment programs for gambling disorder”	Provide access to treatment programs for GD with evidence-based approaches (e.g., cognitive behavioral therapy) and with specialists in GD. Provide specialized programs for marginalized groups (e.g., women with GD), given their unique experience in the field.
	“Make more resources available to let people know about these resources”	Add advertisements for GD treatment options at military bases. Send regular emails about treatment options available to military personnel. Implement a national website for all treatment options and resources available to military personnel that can be filtered for personal needs (i.e., demographics, location, gender) and concern (mental health, physical health, transportation, etc.).
	“On base gambling specialist”	Incorporate a mental health professional specialized in GD to every base and/or have a psychotherapist assigned to each squadron.
	“Peer support groups”	Provide education on resources available through peer support services to those serving in the military (e.g., Battle Buddies). Encourage a community that fosters discussing mental health concerns that arise in the military. Encourage these peer supports to be utilized after services (i.e., as a veteran). Create peer mentorships that include an experienced service member with an incoming service member. Utilize the squad leader as an individual to identify potential risk factors of GD in military personnel.

GD, Gambling disorder; BBGS, Brief Biopsychosocial Gambling Screen.

### Increase awareness

The first theme, *Increase Awareness,* reflects recommendations to educate both military personnel and civilians about gambling risks to promote prevention and facilitate early detection. Participants stressed the value of routine screenings for gambling problems, noting that such practices could reduce stigma and encourage help-seeking (40). Although the DoD has acknowledged the importance of screening (26), many available screening tools have limited diagnostic utility given modest sensitivity and specificity making them unfeasible for most service members (42). We recommend incorporating validated instruments, such as the Brief Biosocial Gambling Screen ([BBGS]; (43), into annual medical evaluations for all service members. These screenings can be conducted periodically throughout service, before, and after service. These screeners can also be implemented online or through paper formats. They are also cost-effective and feasible, as they can be scored by any provider, are relatively short in nature (with the BBGS only being three questions), and are accessible via the internet.

Participants also advocated for mandatory financial education and training on gambling harms, consistent with existing DoD recommendations for addressing substance use and GD (26). Prior research studies suggest that professional training, mental health literacy, and targeted health education can increase help-seeking. Specifically, educational interventions have been shown to lower stigma among military leaders, and online help-seeking programs significantly improve attitudes toward care in both service members and veterans (25, 40). These recommendations are also consistent with public health perspectives on gambling, as experts recommend promoting health education, assessing population health, and recognizing early signs of risk (1).

The structural application of this theme appears to be highly feasible. Prior work has found that an educational intervention decreased help-seeking stigma in military leaders (44) and targeted advertisements resulted in increased help-seeking among veterans (45). Gambling-related education interventions can be added in conjunction to already existing military training and onboarding requirements for service members to increase feasibility. Similarly, research shows that online help-seeking interventions significantly reduce help-seeking stigma in military personnel and veterans (30, 46). Overall, help-seeking interventions in military populations are highly supported for broad mental health concerns; however, military interventions specifically tailored to address PG remain lacking. We recommend interventions to be tailored uniquely to increase help-seeking for gambling concerns in service members and veterans.

While DoD policies encourage disclosure of GD, participants also described severe personal and occupational consequences tied to acknowledging gambling problems (21, 25). These experiences deter disclosure among service members. Increasing awareness and formalizing prevention programs could therefore serve as essential structural changes to enhance help-seeking (40, 47).

### Open door policy

The second theme, *Open Door Policy*, reflects recommendations to create safe spaces for discussing gambling concerns. Participants called for assurances of confidentiality and protections against negative repercussions following disclosure. These recommendations align with treatment guidelines for best practices in substance use treatment for military personnel (48), which emphasize empathetic, patient-centered interactions, and with ethical standards around confidentiality in mental health care (49).

Because the culture of the military is grounded in hierarchy and obedience (50), this environment suppresses disclosure and help-seeking. Specifically, confidentiality in military settings is complicated by policies requiring disclosure for command notification, safety risks, or security concerns (48, 51). Research linking gambling problems to crime (52) and suicidality (53) raises further concerns for national security. This tension creates a difficult balance between ethical obligations and military regulations (54). Some policies have attempted to ensure privacy rights for service members seeking mental health treatment (55); however, the effectiveness of these policies remains unclear (40).

Prior research studies on military mental health providers reveal frequent conflicts between professional ethics and institutional requirements. Providers report strain when navigating their dual roles as clinicians and employees of the military.

Through semi-structured interviews with service members and civilian mental health providers, researchers recommended strategies such as enhanced training in military ethics, structured opportunities for consultation, and a collaborative clinical climate (56). Findings resulted in examples of quality-enhancing behaviors (i.e., behaviors that enhance overall quality of care) that can be implemented by mental health care providers working with military communities. For example, being transparent with service members about dual roles (e.g., explaining limitations of being a provider and also an employee of the military), implementing evidenced-based treatments, being aware of structural and system limitations to client care (e.g., what resources are available, the client’s role within the military, etc.) and reframing client disclosures of information as a way to advocate for the client (e.g., advocating for the client instead of disclosing their gambling concerns) (56).

Implementing such measures could strengthen provider decision making and increase disclosure among service members by cultivating confidentiality and transparency. Discussions and educational trainings for implementing these suggestions can be incorporated in already routinely conducted annual trainings for mental health providers within the military.

Confidentiality is critical for encouraging service members to seek help, yet current policies remain ambiguous and inconsistently applied. We recommend treating GD with the same protections afforded to substance use disorders, clearly communicating confidentiality guidelines to service members, and expanding privacy protections where possible. In addition to reinforcing ethical obligations of confidentiality of mental health care providers within their dual roles to ensure protection of disclosure of gambling concerns. These changes would foster trust, encourage disclosure, and reduce stigma while simultaneously acknowledging operational and security considerations.

### Eliminate gambling and increase leisure

The third theme, *Eliminate Gambling and Increase Leisure*, emphasizes reducing access to gambling opportunities while expanding healthier recreational alternatives. Participants described gambling as a maladaptive (unhealthy) response to boredom and stress (57), reinforced by environments where gambling is normalized (5). In line with public health approaches, participants recommended restricting access (29), limiting advertising (58), and offering more opportunities for leisure and social connection which we posit may decrease service members’ gambling.

The DoD currently provides Morale, Welfare, and Recreation programs (59), but additional targeted activities may be warranted for service members vulnerable to GD. Restricting access to gambling within military bases—particularly slot machines and online sports betting—was a common suggestion and in line with other international governments placing monetary restrictions on wagering (60). Research shows that PG is associated with less regulation of gambling advertisements (61), and time-limits on gambling appear to reduce associated harmful effects (62). Although gambling is prohibited on duty and on government property, overseas bases continue to host slot machines (63), which undermines prevention efforts.

Regulatory changes, combined with expanded recreational programming, could reduce reliance on gambling as a coping mechanism. Future research should examine the unique effects of different types of gambling on the military community (e.g., slot machines vs. sports betting), as recent research is demonstrating increased risk of sports betting in younger individuals (64). Given the evidence linking gambling exposure to harm, stronger restrictions and healthier leisure options appear both feasible and necessary. We suggest providing increased leisure activities while on the base, increased opportunities to connect with outside social support resources, and decreased access to gambling establishments on military bases.

### Increase resources for treatment

The fourth theme, *Increase Resources for Treatment,* captures participants’ recommendations to expand access to gambling-related resources (e.g., new programs, specialized counseling, and peer support). Participants noted the lack of specialized programs and recommended creating new treatment resources, such as Gamblers Anonymous meetings, outpatient clinics, and targeted clinician training while also increasing advertisements for existing sources of support. This recommendation is consistent with DoD guidance (26) emphasizing evidence-based treatment; however, presently there is a total absence of gambling-specific support available to service members. Given that military personnel report that gambling is highly normalized and accessible in the military, the lack of structured gambling-specific support to service members requires further attention (21, 25). Because researchers have identified significant clinical differences between substance use problems and GD in veterans (65), we recommend that military clinicians should be trained in cognitive behavioral therapy, an empirically supported intervention for GD (66), and learn to adapt it to the specific needs of military populations.

Participants also underscored the need for services addressing the experiences of women and other minoritized populations, who face heightened risks (14) and unique challenges for help-seeking (67). Expanding peer support networks was also recommended, given the importance of trust, belonging, and supportive leadership in improving treatment engagement (68, 69). We recommend incorporating peer support services into DoD mental health treatment programming.

These recommendations are highly feasible within existing DoD frameworks (59). Peer support interventions, community-building programs, and reintegration initiatives (e.g., the Yellow Ribbon Reintegration Program) (70) already provide models for integrating GD-specific resources. Consistent with prior work (71), expanding such efforts could increase disclosure, reduce stigma, and improve treatment outcomes.

## Limitations and future research

This study has several limitations. First, findings are based on U.S. service members and veterans and may not generalize to other institutional contexts, although similar patterns have been reported internationally (25). Further studies are needed to explore whether similar patterns emerge In addition, the sample included mostly veterans as opposed to active service members, which could affect the transferability of these recommendations to the current military system. Future research should seek to replicate this study with a larger sample of active service members.

Another limitation of the current study is the lack of cultural diversity among participants. The sample was primarily composed of men (71.4%), White individuals (53.6%), and heterosexual participants (85.7%), limiting the generalizability of these findings to the broader military population. Existing research suggests that sexual, gender, racial, and ethnic minority populations may be uniquely vulnerable to GD (12, 72–74).

Moreover, military personnel with marginalized identities face unique barriers to accessing mental health treatment, including heightened stigma and minority stress (75). Minority stress refers to the chronic distress associated with possessing a marginalized identity that is not affirmed by one’s social environment and may manifest through experiences such as discrimination, stigma, and prejudice (76). Among military populations, minority stress has been associated with reduced treatment-seeking (77, 78), while individuals with marginalized identities may also be at increased risk for gambling-related harms (76). Researchers have suggested that interventions designed to address minority stress by facilitating social connection and engagement with healthcare services, such as the PRIDE in All Who Served program, may reduce internalized stigma and improve mental health outcomes among LGBTQ+ military personnel (41).

These recommendations align with those offered by participants in the present study, particularly the need for peer support and safe spaces in which to discuss gambling-related concerns. However, we did not design the current study to examine the potentially compounding effects of gambling-related barriers to help-seeking among military personnel with marginalized identities. Future research should prioritize amplifying the perspectives of these populations and incorporating questions that capture potentially unique intersectional experiences (e.g., “Have any cultural factors affected your ability to access treatment for problem gambling?”). More broadly, we posit that it is critical that these findings be integrated with ongoing efforts to promote help-seeking among the increasingly diverse population of U.S. military service members and veterans.

Furthermore, future studies should investigate the impact of confidentiality reforms, targeted screening programs, and tailored interventions for GD among military personnel. Greater attention to gambling among vulnerable populations within the military remains essential to address unmet mental health needs as well as to foster greater military readiness for service members.

## Conclusion

Participants provided clear, actionable recommendations to reduce stigma, improve disclosure, and expand support for U.S. service members with GD. These recommendations are consistent with public health principles and could be feasibly implemented through military and federal policy. We recommend that the DoD adopt new legislation mandating: (1) integration of validated screening tools; (2) expanded confidentiality protections; (3) stricter restrictions on gambling access, particularly on overseas bases; (4) increased availability of alternative leisure activities; and (5) development of specialized programs and peer support resources for GD. Although implementation may be challenging (e.g., challenges navigating stigma, confidentiality concerns, and industry pressures), evidence suggests that tailored interventions may monumentally reduce gambling-related harms (30, 45, 46). Adopting these recommendations would represent an invaluable step toward addressing GD as a serious public health issue in the U.S. military.

## Data Availability

The datasets presented in this article are not readily available and may be available upon request. Requests to access the datasets should be directed to Shane Kraus, shane.kraus@unlv.edu.
